# The effects of human umbilical cord-derived mesenchymal stem cell transplantation on endometrial receptivity are associated with Th1/Th2 balance change and uNK cell expression of uterine in autoimmune premature ovarian failure mice

**DOI:** 10.1186/s13287-019-1313-y

**Published:** 2019-07-22

**Authors:** Xueyan Lu, Jingjing Cui, Linlu Cui, Qianqian Luo, Qizhi Cao, Wendan Yuan, Hongqin Zhang

**Affiliations:** 1 College of Basic Medicine, Binzhou Medical University, Yantai, 264003 Shandong China; 2 College of Basic Medicine & Institute of Reproductive Diseases, Binzhou Medical University, Yantai, 264003 Shandong China; 3 The Affiliated Hospital of Binzhou Medical University, Binzhou, 256600 Shandong China

**Keywords:** Human umbilical cord-mesenchymal stem cells, Premature ovarian failure, Endometrial receptivity, uNK cells, Th1/Th2

## Abstract

**Background:**

To explore the mechanism of human umbilical cord-derived mesenchymal stem cell (hUMSC) transplantation to improve ovarian function and the endometrial receptivity in autoimmune premature ovarian failure (POF) mice.

**Methods:**

The POF model was established in mice treated with zona pellucida 3 polypeptide fragment (zona pellucida 3, ZP3). The hUMSCs were transplanted into the POF mice through tail vein injection. Following the transplantation, the serum hormone levels of follicle stimulating hormone (FSH), estrogen (E_2_), progesterone (P), γ-interferon (IFN-γ), interleukin-2 (IL-2), and interleukin-4 (IL-4) were evaluated by ELISA analysis. Morphological changes of ovarian and uterus tissues were examined by HE staining and immunohistochemistry. The expression of Th1/Th2 cytokines of T cells in spleen and CD56^+^CD16^−^ cells (uterine natural killer cells, uNK cells) in uterine was measured by flow cytometry (FCM) and immunohistochemistry. The expression of HOXA10 in uterine endometrium was examined by immunohistochemistry and RT-PCR analysis. The pinopodes of epithelial cells in uterine endometrium were examined by scanning electron microscopy.

**Results:**

Following hUMSC transplantation, the serum levels of E_2_, P, and IL-4 were increased but FSH, IFN-γ, and IL-2 levels were decreased in POF mice. Also, the transplantation of hUMSCs caused an increase in total number of healthy follicles and decrease of atresia follicles. The expression of HOXA10 gene was significantly increased but the CD56^+^CD16^−^ uNK cells decreased in the endometrium of uterine. The ratio of Th1/Th2 cytokines was also significantly decreased.

**Conclusion:**

The data suggest that the recovery of ovarian function and endometrial receptivity in POF mice was regulated by the balance of Th1/Th2 cytokines and expression of uNK cells in the endometrium following hUMSC transplantation.

## Background

POF is one of the common disorders found in women’s reproductive, which is characterized by high gonadotropin expression, low estradiol expression, and follicular dysplasia. The causes are unknown and have been reported with many complicated factors such as genetic defects, infection, autoimmunity, chemotherapy injury, and other factors [1–5]. Among them, the autoimmune factors may account for 5–30% [6, 7]. Due to the complexity of POF, there is no completely effective therapy method to treat this disease. Recently, stem cell therapy has gained great interest as a promising approach to treat POF. Some papers and our previous studies have shown that mesenchymal stem cell transplantation can significantly restore the ovarian function and follicle development in autoimmune-induced POF mice. However, the exact mechanism on how mesenchymal stem cell transplantation restores the ovarian function is still unclear. As reported, the follicular development, endometrial receptivity and maternal-fetal interaction are three key factors for successful pregnancy. Among them, dysfunction of any factor might lead to infertility [8]. In the current study, the investigation on the mechanism of hUMSC transplantation to treat POF mice has focused on how it recovers ovarian function and affects the endometrial receptivity in uterine.

Endometrial receptivity [9] refers to the ability of the endometrium to allow implantation of an embryo during a specific period. Embryo implantation is similar to allotransplantation, which is also a complex process involving many immune regulation factors such as the imbalance of Th1/Th2 cytokines. Uterine natural killer (uNK) cells play an important role in the maintenance of Th1/Th2 cytokine balance in the endometrial local immune response. The uNK cells are a group of special cells in endometrial stroma that the majority of uNK cell phenotype is stained as CD56^+^CD16^−^ [10–12]. The uNK cells increase rapidly during the secretory phase and early pregnancy. If uNK cells are activated by some pathological factors, the cells will secrete cytotoxic factors to result in the imbalance of Th1/Th2 cytokines. Subsequently, this enhances the killing effect of Th1 cytokines to cause endometrial receptive ability reduction and embryo implantation failure [13, 14]. As reported, the changes in endometrial receptivity depend on the spatiotemporal specific expression of some particular set of genes in the endometrium. Among them, the HOXA10 gene has been considered as a molecular marker to measure endometrium receptivity [15].

Some studies have found that a large number of immune cells infiltrate into the ovarian tissue of in mice with premature failure, which include T lymphocytes [16, 17], B lymphocytes, and natural killer cells [18]. The literature also reported that the balance of Th1/Th2 cytokine expression in T lymphocytes subgroup is disordered in patients with POF [19]. Therefore, the goal of the study is to investigate if the immune regulation on Th1/Th2 cytokine balance and uNK cell expression are involved in the ovarian function recovery and endometrial receptivity improvement following hUMSC transplantation in POF mice.

## Methods

### Experimental animals

Female mice (Balb/c) at the age of 8 weeks were purchased from Jinan Pengyue Experimental Animal Breeding Co. Ltd. (Shandong, China).

### Isolation and culture of hUMSCs

The use of human umbilical cord tissue was approved by the Institutional Ethics Committee. The collected umbilical cord was cut into small pieces. The pieces with a diameter of less than or equal to 1.5 mm were collected after the filtration through a coarse filter. The isolated tissues were incubated with low-glucose Dulbecco’s modified Eagle’s medium (Gibco), and the adherent cells were used for the treatment after the confluence reached 80% following three generations. Cell differentiation was examined using alizarin red staining to identify cell osteogenicity; Oil red O staining was used to examine cell lipogenicity. The fourth generation of cells was used to make single-cell suspension for the experiment. Flow cytometry (FCM) was conducted to detect the cell surface and intracellular markers such as CD19, CD73, CD90, CD34, HLA-DR, and CD44 (Invitrogen) [20].

### POF mouse model establishment

The ZP3-induced POF mouse model was established according to the literature report [21, 22]. The amino acid sequence of the murine ZP3_330–342_ peptides used in this study is NSSSSQFQIHGPR. Adult mice (*n* = 80) were randomly divided into four groups (*n* = 20 each): control group, POF group, POF + PBS group, and POF + hUMSCs group. No any treatment was performed in the control group. The mice in the POF group were injected subcutaneously with 0.15 ml 50 nmol/L of ZP3 emulsified in complete Freund’s adjuvant (CFA) after feeding for 1 week. Seven days after the first injection, the mice were subcutaneously injected with 0.15 ml 50 nm/L of ZP3 emulsified Freund’s incomplete adjuvant (FIA). In the hUMSC treatment groups, 1 × 10^6^ hUMSCs [23–25] were injected via tail vein on day 14 after the second treatment. The POF + PBS group was injected with the same volume of PBS by tail vein. Two weeks after hUMSC transplantation, the mice were euthanized. The blood and ovary tissues were collected for further experiment.

#### Hormone and cytokine measurement in serum

At the necropsy, blood samples were collected and centrifuged at 4000 rpm for 10 min. The serum levels of hormone such as follicle stimulation hormone (FSH), estradiol (E2), progesterone (P), and cytokines of γ-interferon (IFN-γ), interleukin-2 (IL-2), and interleukin-4 (IL-4) were measured by ELISA assay kits (Mlbio, China) according to the manufacturer’s instructions.

### Analysis of Th1/Th2 subtype in spleens and uNK cell expression in uterine by FCM

Lymphocytes were isolated from the spleens with lymphocyte separation solution. The isolated cells were washed and resuspended in PBS. Then, the cells were incubated with T cell surface marker monoclonal antibodies of anti-mouse CD3-APC and CD4-FITC at 4 °C for 10 min in the dark. After washing, the cells were incubated with anti-mouse IFN-γ-PerCP-cy5.5 and IL-4-PE (eBioscience, San Diego, USA) for flow cytometry analysis.

According to the vaginal smear results, the uterine tissue at secretory phase was collected and uNK cells were isolated with lymphocyte separation solution. The isolated cells were washed and resuspended in PBS. The phenotype of cells was analyzed by flow cytometry after being incubated with anti-mouse CD3-APC, CD56-PE, and CD16-FITC (eBioscience) monoclonal antibodies at 4 °C for 10 min in the dark.

### Morphological changes in ovary and uterus tissues

The isolated ovary and uterus tissues in paraffin sections were prepared after fixation, dehydration, and implantation with thickness of 4 μm and routine HE staining. The morphological changes of ovaries were observed under the microscope. The number of follicles at all stages was counted [26]. The uterus at secretory stage was collected by vaginal smears for routine HE staining and examination.

### Evaluation of HOXA10 protein expression in endometrial tissue

The collected endometrial tissues from treatment and control mouse group were fixed and cut into sections (4 μm) and then incubated with rabbit primary anti-HOXA10 polyclonal antibody (1:500 dilution; Bioss) at 4 °C overnight. After that, incubate with secondary antibody at 37 °C for 30 min. Diaminobenzidine (DAB) was used as a chromogen for color reaction. The cells stained as brown granules in cytoplasm were considered HOXA10 expression-positive cells. The staining intensity (SI) was scored on the following scale: no stain as 0, light yellow as 1, tan color as 2, and brown color as 3. According to the percentage of positive (PP) staining cells, the point of positive cell degree was graded based on the percentage: 0 point (PP < 5%), 1 point (PP = 5–25%), 2 points (PP = 25–50%), 3 points (PP = 50–75%), and 4 points (PP > 75%). The final IRS score is calculated by multiplying SI and PP as literature reported [26].

### Hoxa10 mRNA expression in endometrial tissue

The Hoxa10 mRNA expression in the endometrium of the uterus was measured by real-time polymerase chain reaction (RT-PCR). Total RNA was extracted from the uterine tissues. The amount of RNA was quantitated by spectrophotometry. RT-PCR was conducted according to the manufacturer’s instruction. The primer sequences of Hoxa10 and Gapdh include the upstream primer (5′-TTCGCCGGAGAAGGACTC-3′, 5′-GCCTTCCGTGTTCCTACCC-3′) and the downstream primer (5′-TCTTTGCTGTGAGCCAGTTG-3′, 5′-TGCCTGCTTCACCACCTTC-3’). The reaction, containing SYBR, primers (forward and reverse), 5 μl of cDNA, ddH_2_O, were done by using Fast Start Universal SYBR Green Master (ROX) (Roche, Germany). The experimental data were normalized by endogenous housekeeping gene glyceraldehyde-3-phosphate dehydrogenase (Gapdh) expression value.

### CD56, IL-4, and IFN-γ expression in the uterine endometrium

The expression of CD56, IL-4, and IFN- γ in the isolated uterine tissues were examined by immunofluorescence. The sections of uterine tissues were incubated with fluorescent antibodies CD56 (1:100, eBioscience), IL-4 (1:100, eBioscience), and IFN-γ (1:50, eBioscience) overnight at 4 °C. After incubation, the nucleus was stained with DAPI and examined by laser scanning confocal microscope.

### Pinopode examination of endometrial epithelial cells

The epithelial cells were isolated and fixed with 2.5% glutaraldehyde and 1% osmium tetroxide. After being dehydrated by gradient alcohol, the pinopodes of endometrial epithelial cells were examined by scanning electron microscopy (SEM).

### Statistical analysis

Data were represented by mean ± SD, SPSS 16.0 statistical software was used, and a one-way analysis of variance (ANOVA) was used for statistical analysis. *P <* 0.05 was considered statistically significant.

## Results

### hUMSC characterization

**Fig. 1:**
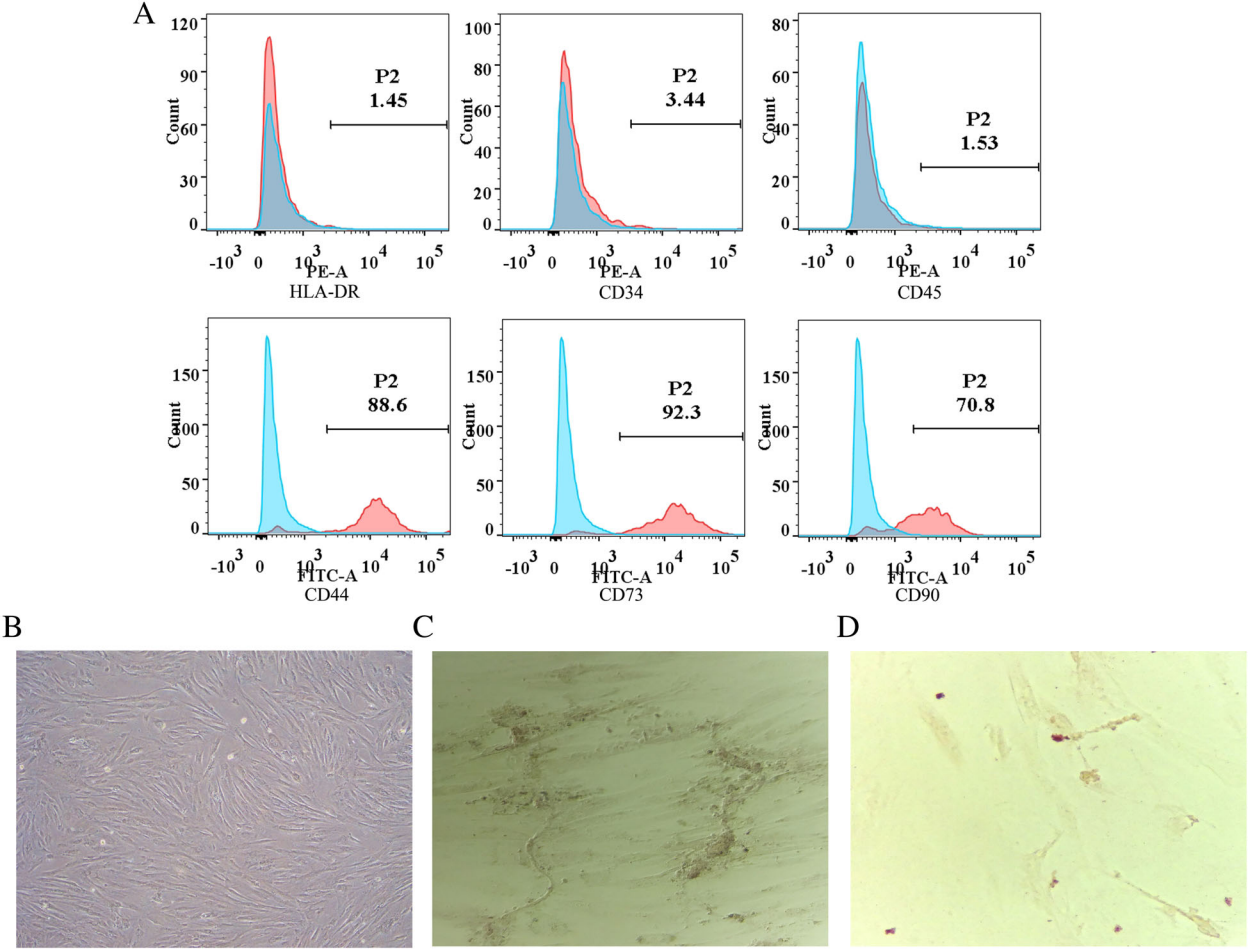
Cell phenotype and differentiation characterization in hUMSCs. Cell phenotype was characterized with specific surface markers using flow cytometry analysis. Cell differentiation was confirmed by cellular staining. **a** Blue histograms represent isotype control staining, and red histograms represent the expression of specific cell surface markers. **b**–**d** Cells were cultured under conditions to differentiate into osteoblast or adipogenesis cells. **b** The fibroblast-like morphology of primary hUMSCs. **c** Osteoblasts with alizarin red staining; positive staining was shown brown color with calcium deposition (× 400). **d** Adipogenic cells with the accumulation of neutral lipid vacuoles which are stained with Oil Red O (× 400). hUMSC human umbilical cord-derived mesenchymal stem cells

After primary culture of hUMSCs for 7–10 days, the adherent cells grow into long fusiform cells with fibroblast-like morphology. In passage 2, the image of spindle-shaped cells is shown in Fig. 1b. The immunophenotype of the cells was characterized by flow cytometry with cell-type-specific biomarkers. As shown in Fig. 1a, the cells with positive staining are CD44 (88.6%), CD73 (92.3%), and CD90 (70.8%), and the cells with negative staining for markers HLA-DR (1.45%), CD34 (3.44%), and CD45 (1.53%). The cells were differentiated into two types of cells (Fig. 1c, d): osteogenic and adipogenic phenotypes. The differentiation capacity of hUMSCs towards the chondrogenic was demonstrated by alizarin red staining. The adipogenic cells were stained in Oil Red with the accumulation of neutral lipid vacuoles.

### Serum reproductive system hormone changes in POF mice following hUMSC transplantation

**Fig. 2:**
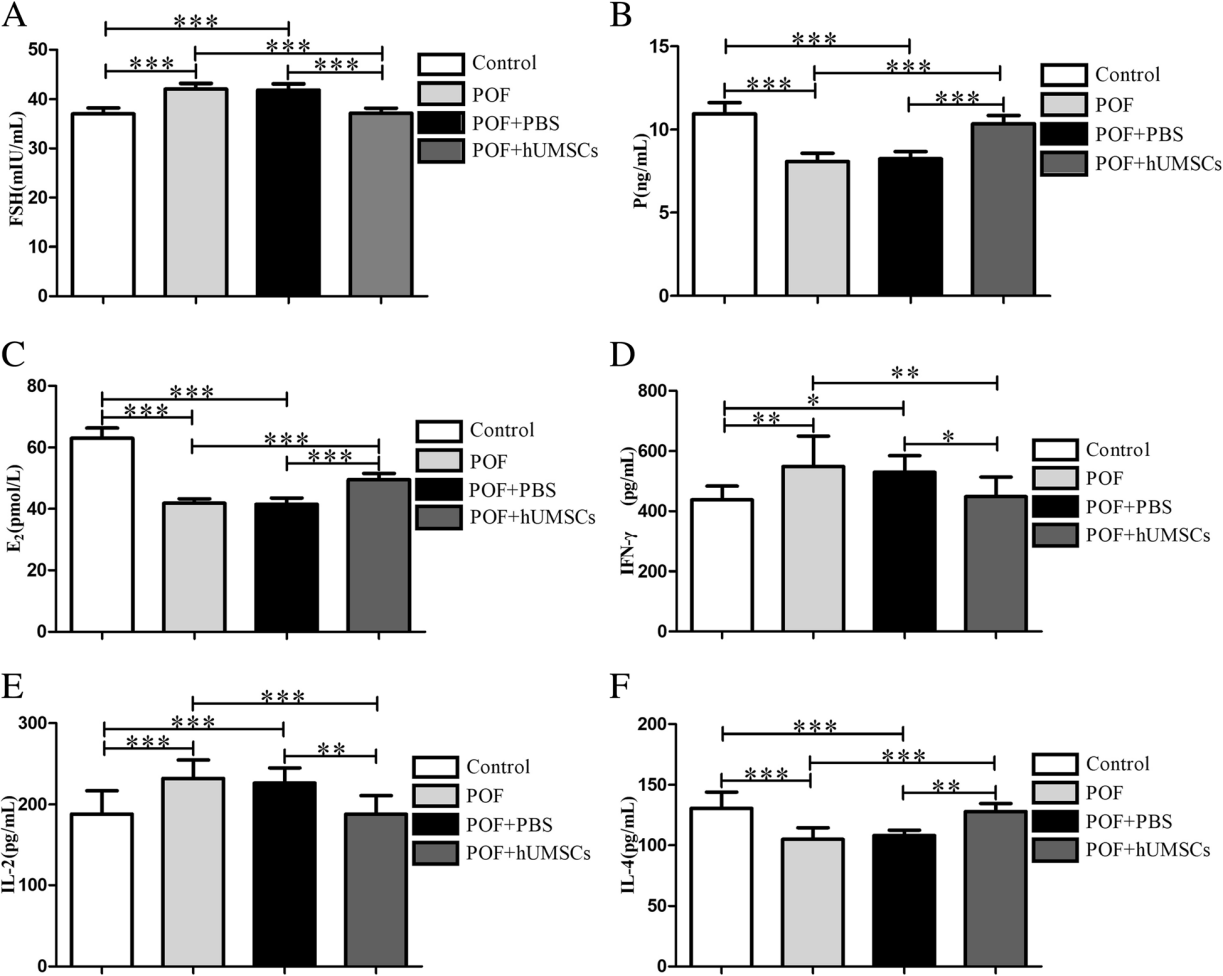
Effects of hUMSC transplantation on serum hormone release in POF mice. The serum levels of hormones were measured by ELISA. **a** FSH. **b** P. **c** E_2_. **d** IFN-γ. **e** IL-2. **f** IL-4. Data are presented as mean ± SD. *P < 0.05, **P < 0.01, ***P < 0.001. ELISA enzyme-linked immunosorbent assay, FSH follicle stimulating hormone, P progesterone, E_2_ estradiol, IFN-γ γ-interferon, IL-2 interleukin-2, IL-4 interleukin-4

To evaluate the ovary function in POF mice following hUMSC transplantation, the serum levels of reproductive system hormones were examined. The amounts of E_2_, P, and FSH were measured by ELISA. The results showed that the serum levels of E_2_ and P in the POF mice were significantly lower than the control group, but the levels of FSH were significantly increased (*P <* 0.001). Following hUMSC transplantation, the E_2_ and P levels were significantly increased with a decrease of FSH as shown in Fig. 2.

### Effects of hUMSC transplantation on immunoregulatory molecule production in POF mice

To determine if hUMSC transplantation affects the cytokine release, IL-2 and IFN-γ secreted by Th1 cells and IL-4 secreted by Th2 cells were measured by ELISA. The results showed that the serum levels of IFN-γ and IL-2 were significantly increased but IL-4 was decreased in POF mice compared to the control group (*P <* 0.01). After hUMSC transplantation, the amounts of these cytokines were reversed (Fig. 2). The higher levels of IFN-γ and IL-2 in POF mice were decreased, but the IL-4 levels were increased (*P <* 0.01).

### Effects on the Th1/Th2 cell produced cytokines in spleen and uNK cell number changes in the uterus following hUMSC transplantation in POF mice

**Fig. 3:**
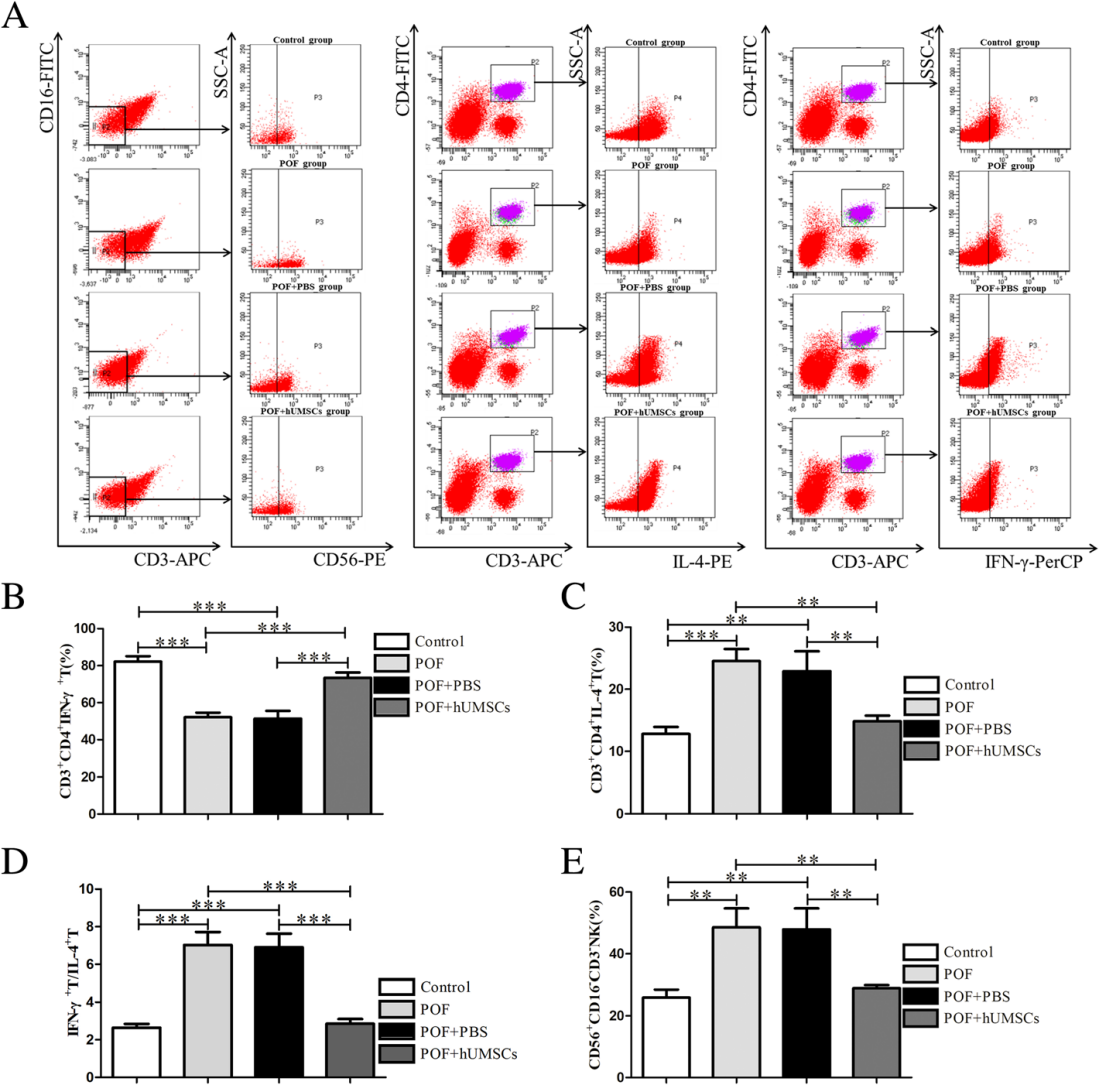
Expression of IL-4 and IFN-γ cytokines in spleen and uNK cell numbers in uterus following hUMSC transplantation. **a** Spleen and uterine tissues were stained with specific cell surface markers to identify the uNK cells and measure IL-4/IFN-γ expression by flow cytometry. **b**–**e** Quantitative analysis on the release of Th1 cytokine (IFN-γ), Th2 cytokine (IL-4), Th1/Th2 ratio, and uNK cells, respectively. Data are presented as mean ± SD. *P < 0.05, **P < 0.01, ***P < 0.001. hUMSC human umbilical cord-derived mesenchymal stem cells, IFN-γ γ-interferon, IL-4 interleukin-4, uNK uterine natural killer

Based on the vaginal smear results, the uterine tissue at the secretory phase was collected. The cells were isolated with lymphocyte separation solution and were analyzed by flow cytometry. Compared with the control group, higher percentage of the uNK cells was found in the uterus of POF mice. After hUMSC transplantation, the number of uNK cells was significantly reduced in POF mice. In the spleen, the elevated IFN-γ expression in the POF group was decreased after hUMSC transplantation (*P <* 0.001). In contrast, the reduced IL-4 expression in POF mice was reversed after hUMSC transplantation (Fig. 3).

### Changes of ovary morphology and follicle numbers following hUMSC transplantation in POF mice

**Fig. 4:**
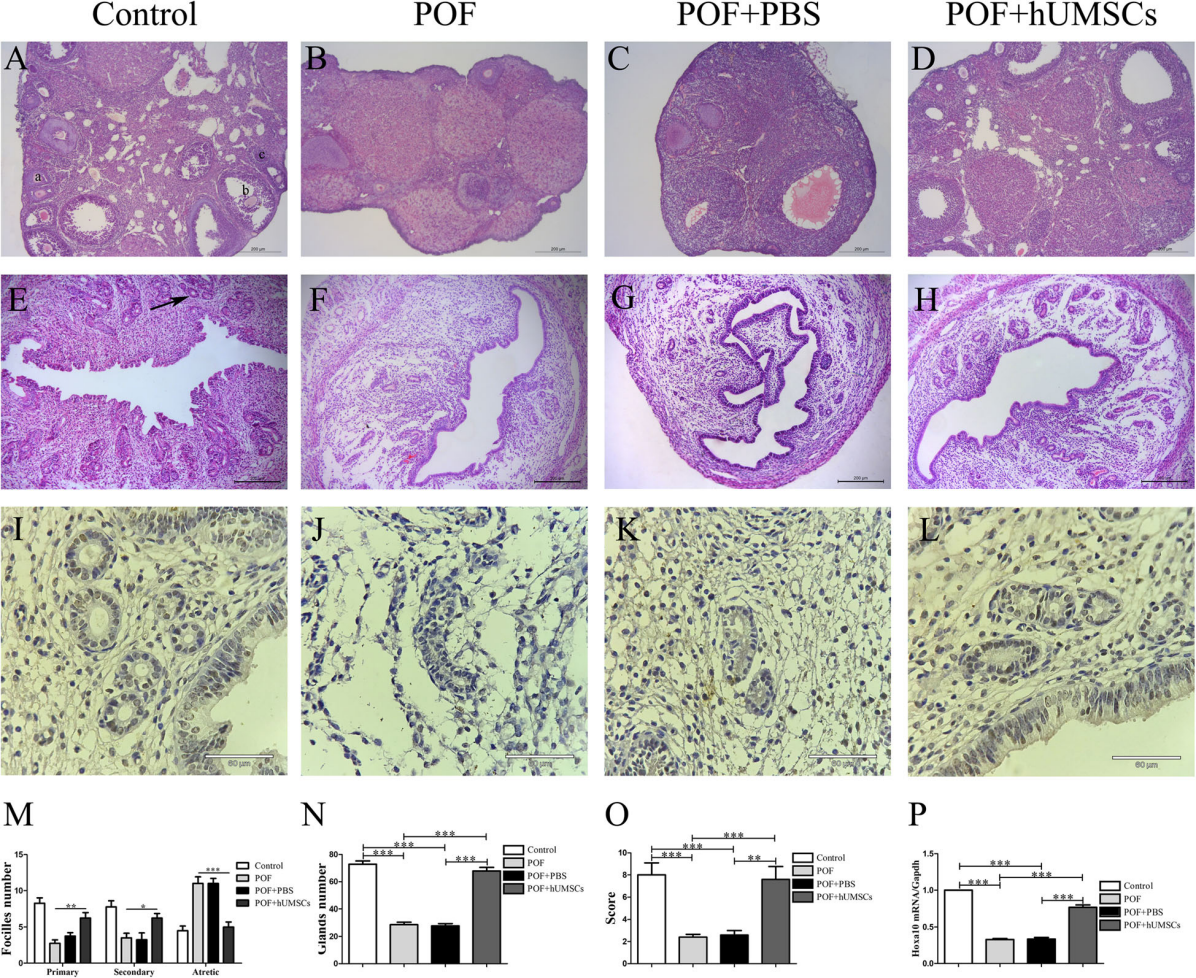
Effects of hUMSC transplantation on ovary tissues morphology, uterus morphology, and HOXA10 activation in uterus of POF mice. **a**–**d** Histopathological examination of ovarian tissues in each group mouse. Different stages of follicles: **a** primary follicles, **b** secondary follicles, and **c** atretic follicles. **m** Summary of ovarian follicle numbers in each group. **e**–**h** Histopathological examination of uterus tissues of each group (the black arrow indicates a gland). **n** Uterus gland count in each group. Representative HOXA10 staining with tan particles in cells of each group. **o** Summary of HOXA10 protein expression scores in the uterus tissues. **p** The quantitative analysis of HOXA10 mRNA expression in uterus tissues. Data are presented as mean ± SD. *P < 0.05, **P < 0.01, ***P < 0.001. POF premature ovarian failure, hUMSCs human umbilical cord-derived mesenchymal stem cells

Through histopathological examination, a large number of follicles including functional follicles (primitive follicles, primary follicles, secondary follicles) and atresia follicles are present in ovary tissues of the control group. In contrast, the lower mass, abnormal structural, interstitial hyperplasia, severe fibrosis, and less number of functional follicles were observed in the ovary tissues of POF mice. The number of atresia follicles in POF mice was significantly increased as compared to the control group (*P <* 0.001). After hUMSC transplantation, the morphology and structure of the ovary tissues showed recovery with the evidence of decreased fibrosis and increased number of functional follicles at all developmental stages. Also, the closed follicles were significantly decreased as shown in Fig. 4.

### Effects of hUMSC transplantation on uterus morphology and gland numbers in POF mice

At the necropsy, the uterus tissues were collected for histopathological examination. In the control group, the endometrium was shown mature with loose interstitial vessels and abundant glands. In comparison, the endometrium in POF mice was poorly developed with lesser blood vessel in the interstitium. The gland numbers were much less, and the development of glandular dysplasia was relatively simple. Following hUMSC transplantation, the number of glands and vessels were significantly increased as shown in Fig. 4.

### Effects of hUMSC transplantation on HOXA10 protein expression in endometrial tissue of POF mice

To evaluate the endometrium receptivity in POF mice and immunohistochemical staining of endometrial, the results showed that the positive signals of HOXA10 expression were present in the cytoplasm (tan particles) of the endometrium in all group mice. When compared with the control group, the expression of HOXA10 protein in the POF group was significantly reduced (*P <* 0.01). However, following hUMSC transplantation, the expression of HOXA10 in POF mice was increased as shown in Fig. 4i–l. The similar results were observed in HOXA10 mRNA expression by PCR analysis.

### CD56, IL-4, and IFN-γ expression in the endometrium following hUMSC transplantation in POF mice

**Fig. 5:**
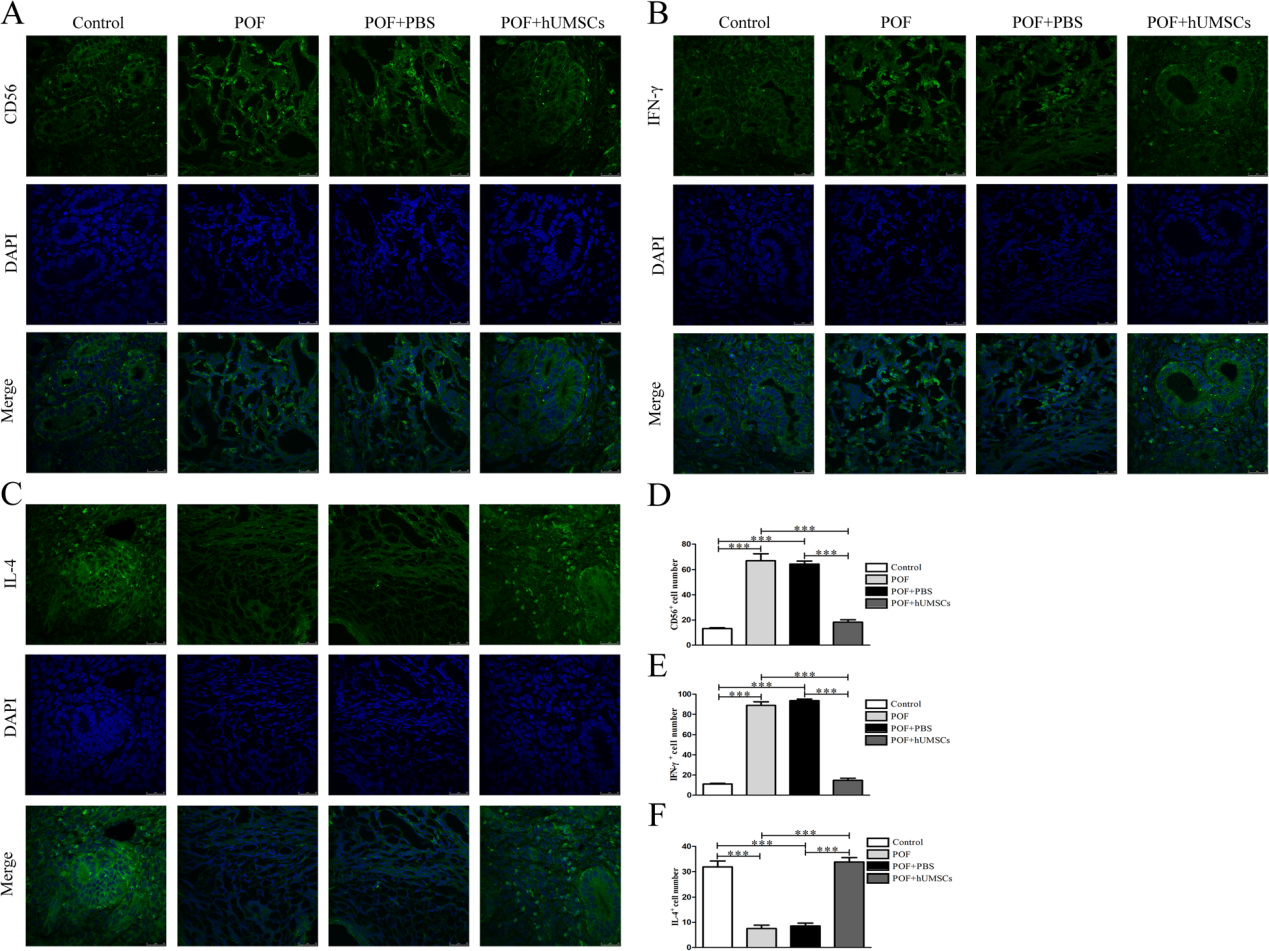
Immunofluorescence detection on CD56, IL-4, and IFN-γ expression in endometrium of POF mice following hUMSC transplantation. **a** CD56 expression. **b** IFN-γ expression. **c** IL-4 expression. **d** CD56-positive cell count. **e** IFN-γ-positive cell count. **f** IL-4-positive cell count. Data are presented as mean ± SD. ***P < 0.001. IFN-γ γ-interferon, IL-4 interleukin-4

The expressions of CD56, IFN-γ, and IL-4 in the endometrium were examined by immunofluorescence. As shown in Fig. 5, the hUMSC transplantation caused a decreased expression of CD56 and IL-4 in POF mice. In comparison, the IFN-γ expression was significantly increased after hUMSC transplantation. The similar results were observed in the CD56, IFN-γ, and IL-4 expression in the endometrium by flow cytometry as shown in Fig. 3.

### Effect of hUMSC transplantation on pinopodes of endometrial epithelial cells in POF mice

**Fig. 6:**
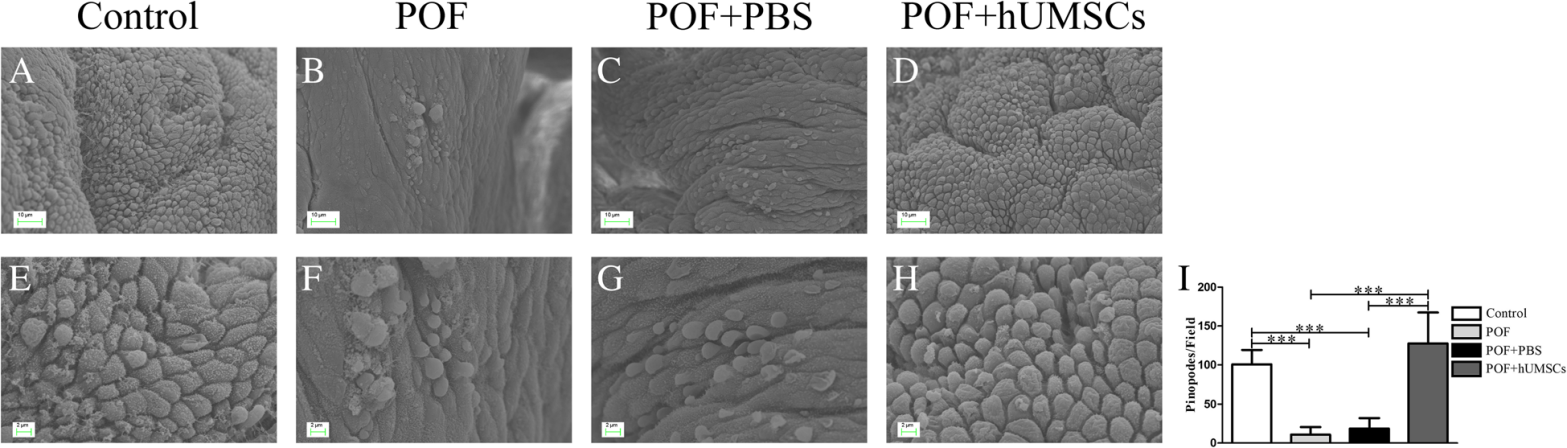
Effects of hUMSC transplantation on pinopodes changes of endometrial epithelial cells in POF mice. The pinopodes were observed by scanning electron microscopy (**a** to **d**: 1.00KX, **e** to **h**: 3.00KX). **i** Summary of the total numbers of pinopodes in endometrial epithelial cells per field. Data were presented as mean ± SD. ***P < 0.001. hUMSCs human umbilical cord-derived mesenchymal stem cells, POF premature ovarian failure

The pinopodes of endometrial epithelial cells were examined by scanning electron microscopy. In the control group, the endometrial surface was regular with abundant expression of pinopodes. The morphology and size of pinopodes were similar with clear boundaries. In comparison, less amount of pinopodes were observed in the endometrium of POF mice. Also, the pinopodes showed various morphologies and sizes without obvious microvilli. The cell boundaries are unclear. Following the hUMSC transplantation, the endometrial surface became more regular with plenty of microvilli on the cell surface. The intercellular space was clearly observed as shown in Fig. 6.

## Discussion

In recent years, stem cell transplantation has gained great attention in repairing injured ovarian tissues and recovering the ovarian function. In our previous study, the data has shown that mesenchymal stem cells transplantation can significantly recover the ovarian function in POF mice induced by ZP3 injection [27, 28]. However, the exact mechanisms are still unclear. In the current study, we further investigated the immune regulatory mechanism of hUMSC transplantation on ovarian function and uterine endometrial receptivity in autoimmune POF mice. The recovery of ovarian function following hUMSC transplantation was demonstrated by the restoration of normal estrus cycle, increased growth of follicles, and restored hormone level of FSH and E_2_. The scanning electron microscopy also demonstrated the increased pinopodes in the endometrium, which suggested the improved receptivity status of the endometrium in the uterus. The increased receptivity of the endometrium was also supported by the expression of HOXA10 in the endometrium.

As reported, the HOXA10 gene has been considered a molecular marker to measure endometrium receptivity and plays an important role in the establishment of endometrium receptivity, the formation of cytoplasm, implantation of embryos, and stability of maternal immune interface [29, 30]. The expression of HOXA10 protein in the endometrium changes periodically with the menstrual cycle and is significantly upregulated after the middle stage of secretion. At the middle and late stages of secretion, the expression level reaches its peak, which is consistent with the embryo implantation window [31–33] and the increase of progesterone in the blood [34]. In our study, the immunohistochemistry and PCR analysis demonstrated that the hUMSC transplantation significantly increased the expression of HOXA10 in the secretory endometrium of POF mice. The data further support the recovery of endometrium receptivity in POF mice after hUMSC treatment.

Some studies have shown that the decrease of endometrial receptivity is related to immunoregulatory dysfunction at the local maternal-fetal interface. The balance of Th1/Th2 cytokines plays an important role in the regulation of its immune function [35–37]. The endometrial uNK cells, macrophages, dendritic cells, and T cells are involved in maintaining the immune cytokine balance [38, 39]. In addition, more and more studies have focused on the endometrial NK cells (uNK) and its related factors. It is reported that uNK cells play an important role in the endometrial local immune [40], and the failure of embryo implantation was related to the increase of uNK cells and the immune disorder caused by Th1 cytokines release [41]. The uNK cells are mainly distributed in endometrial stromal and glandular epithelium with a special surface marker CD56^+^CD16^−^. The number of uNK cells reached its peak after the middle stages of secretion until the early stages of pregnancy in the endometrium, and the number of uNK cells gradually increased to about 90% of the total number of endometrial lymphocytes [42]. The change of the numbers is regulated by the increase of estrogen and progestogen. Mature uNK cells contain a large number of particles with a strong secretion function and can secrete a variety of cytokines such as IL-2 and IFN-γ [43]. The secretion function of uNK is considered the main mechanism to regulate the balance of cytokine release in the endometrium of pregnancy [44, 45].

The serum cytokines secreted by T cells play an important role in the regulation of the immune system. The IL-2 and IFN-γ cytokines are mainly secreted by Th1 cells to promote cellular immunity, and IL-4 cytokine secreted by Th2 cells is involved in the humoral immunity [46, 47]. In the current study, the data show that the hUMSC transplantation not only changed the release of Th1/Th2 cytokines in the serum but also in the endometrium with a similar trend as the uNK cells. This suggests that the changes of Th1/Th2 cytokine production may be mediated by the uNK cells in secretory endometrium. The immunoregulation plays an important role in the recovery of endometrial injury and endometrium receptivity in the uterus of POF mice after hUMSC transplantation.

As we know, Th1 cytokines are involved in graft rejection and Th2 cytokines are involved in immune tolerance during pregnancy. The maternal-fetal interface of normal pregnancy is mainly dominated by Th2 cytokines which secret IL-4 cytokine to inhibit the activation of IL-2. The pro-inflammatory cytokine IFN-γ is secreted by T cells and uNK cells [48]. Studies have shown that IL-2 and IFN-γ secreted by Th1 cells have an inhibitory effect on the secretion of Th2 cytokines, while IL-4 secreted by Th2 cells can inhibit the production of Th1 cytokines [49]. The regulation among cytokines forms a special cytokine network in the immune microenvironment to maintain the dynamic balance of normal pregnancy. In addition, IFN-γ may be involved in the process of follicular atresia. It acts as an initiator to enhance MHC I and MHC II of ovarian granulosa cell (GC)-induced follicular atresia. The increased Th1/Th2 ratio leads to the disorder of E_2_ and P secretion and results in the failure of periodic cycling in the endometrium and reduction of endometrial receptivity. The data in our study suggest that the regulation on the ratio of Th1/Th2 cytokines and the secretion of uNK cells may be involved in the ovarian function recovery and endothelium receptivity in POF mice following hUMSC transplantation. This provides useful information to develop new pharmaceutical therapies to patients with POF failure in the future.

## Conclusion

The results from our study showed that hUMSC transplantation can recover the function of impaired ovarian and endothelium receptivity in POF mice, which is mediated by the change of Th1/Th2 cytokine ratio.

## Data Availability

All data generated and/or analyzed during this study are included in this published article.

## Abbreviations

CFA: Complete Freund’s adjuvant
DMEM: Dulbecco’ s modified Eagle’ s medium
E_2_: Estradiol
ELISA: Enzyme-linked immunosorbent assay
FCM: Flow cytometry
FIA: Freund’s incomplete adjuvant
FSH: Follicle stimulating hormone
GCs: Granulosa cells
hUMSCs: Human umbilical cord-derived mesenchymal stem cells
IFN-γ: γ-Interferon
IL-2: Interleukin-2
IL-4: Interleukin-4
P: Progesterone
POF: Premature ovarian failure
RT-PCR: Real-time polymerase chain reaction
SEM: Scanning electron microscopy
uNK: Uterine natural killer cells
ZP3: Zona pellucida 3 peptide
